# Event-related potentials to unattended changes in facial expressions: detection of regularity violations or encoding of emotions?

**DOI:** 10.3389/fnhum.2013.00557

**Published:** 2013-09-11

**Authors:** Piia Astikainen, Fengyu Cong, Tapani Ristaniemi, Jari K. Hietanen

**Affiliations:** ^1^Department of Psychology, University of JyväskyläJyväskylä, Finland; ^2^Department of Mathematical Information Technology, University of JyväskyläJyväskylä, Finland; ^3^Human Information Processing Laboratory, School of Social Sciences and Humanities, University of TampereTampere, Finland

**Keywords:** equiprobable condition, facial expressions, independent component analysis, oddball condition, visual mismatch negativity

## Abstract

Visual mismatch negativity (vMMN), a component in event-related potentials (ERPs), can be elicited when rarely presented “deviant” facial expressions violate regularity formed by repeated “standard” faces. vMMN is observed as differential ERPs elicited between the deviant and standard faces. It is not clear, however, whether differential ERPs to rare emotional faces interspersed with repeated neutral ones reflect true vMMN (i.e., detection of regularity violation) or merely encoding of the emotional content in the faces. Furthermore, a face-sensitive N170 response, which reflects structural encoding of facial features, can be modulated by emotional expressions. Owing to its similar latency and scalp topography with vMMN, these two components are difficult to separate. We recorded ERPs to neutral, fearful, and happy faces in two different stimulus presentation conditions in adult humans. For the oddball condition group, frequently presented neutral expressions (*p* = 0.8) were rarely replaced by happy or fearful expressions (*p* = 0.1), whereas for the equiprobable condition group, fearful, happy, and neutral expressions were presented with equal probability (*p* = 0.33). Independent component analysis (ICA) revealed two prominent components in both stimulus conditions in the relevant latency range and scalp location. A component peaking at 130 ms post stimulus showed a difference in scalp topography between the oddball (bilateral) and the equiprobable (right-dominant) conditions. The other component, peaking at 170 ms post stimulus, showed no difference between the conditions. The bilateral component at the 130-ms latency in the oddball condition conforms to vMMN. Moreover, it was distinct from N170 which was modulated by the emotional expression only. The present results suggest that future studies on vMMN to facial expressions should take into account possible confounding effects caused by the differential processing of the emotional expressions as such.

## Introduction

Other people's facial expressions convey socially important information about other individuals' emotions and social intentions (Keltner et al., 2003). Therefore, it is not surprising that facial expressions are, among other biologically and socially significant information, processed automatically and rapidly in the brain (e.g., Adolphs, 2002; Palermo and Rhodes, 2007).

Because of its good time resolution, measurement of event-related potentials (ERPs) has been widely used in studies investigating the early stages of facial information processing. An ERP component called visual mismatch negativity (vMMN; visual counterpart of mismatch negativity, defined originally in the auditory modality, Näätänen et al., 1978; for a review see Näätänen et al., 2010) is a feasible method to study automatic encoding of several types of visual stimuli including faces. vMMN is elicited to rare stimuli (“deviant”) interspersed with repeated (“standard”) stimuli and observed as a differential ERP response between these two. vMMN can be observed in conditions where the participants are instructed to ignore the visual stimuli eliciting the vMMN and attend to other visual stimuli (e.g., Stefanics et al., 2012) or auditory stimuli (e.g., Astikainen and Hietanen, 2009). In addition to changes in low-level visual features, such as orientation of a bar (e.g., Astikainen et al., 2008) or color (e.g., Czigler et al., 2002), it has also been associated with changes in complex visual features, including human hands (Stefanics and Czigler, 2012) and facial expressions (Susac et al., 2004; Zhao and Li, 2006; Astikainen and Hietanen, 2009; Chang et al., 2010; Kimura et al., 2011; Li et al., 2012; Stefanics et al., 2012).

vMMN is considered to reflect a process of detecting a mismatch between the representation of the repeated standard stimulus in transient memory and the current sensory input (Czigler et al., 2002; Astikainen et al., 2008; Kimura et al., 2009) similarly to auditory MMN (for the trace-mismatch explanation of MMN, see Näätänen, 1990). The standard stimuli can also be physically variant, but if they form sequential regularity, deviant stimuli violating this regularity elicit vMMN (Astikainen and Hietanen, 2009; Kimura et al., 2010, 2011; Stefanics et al., 2011, 2012; for a review see Kimura, 2012). For example, serially presented pictures of faces can be of different identities, but a vMMN is elicited if, say, rare fearful faces are interspersed among emotionally neutral faces, suggesting that a representation of a “neutral face” can be abstracted among several low-level features (Astikainen and Hietanen, 2009). Along the same lines, vMMN elicitation has recently been linked to the predictive coding theories (Friston, 2005), which postulate a predictive error between the neural model based on the representations of visual objects in memory and the actual perceptual input (Winkler and Czigler, 2012).

vMMN to facial expressions has been reported at different latency ranges, starting from 70 up to 360 ms post-stimulus (Susac et al., 2004; Zhao and Li, 2006; Astikainen and Hietanen, 2009; Chang et al., 2010; Kimura et al., 2011; Li et al., 2012; Stefanics et al., 2012), and sometimes multiple responses with different latencies have been reported (Astikainen and Hietanen, 2009; Chang et al., 2010; Li et al., 2012; Stefanics et al., 2012). Nevertheless, a consistent finding has been a vMMN observed around the same latency (~130–200 ms after stimulus onset) and in the same scalp location (parieto-occipital region) with the well-known face-sensitive N170 response (Zhao and Li, 2006; Astikainen and Hietanen, 2009; Chang et al., 2010; Stefanics et al., 2012). The N170 was originally associated with the structural encoding of faces (Bentin et al., 1996), but several studies have shown its sensitivity to emotional expressions (Batty and Taylor, 2003; Eger et al., 2003; Caharel et al., 2005; Williams et al., 2006; Blau et al., 2007; Leppänen et al., 2007; Schyns et al., 2007; Japee et al., 2009; Vlamings et al., 2009; Wronka and Walentowska, 2011, for the studies showing no emotional modulation of N170, see Eimer and Holmes, 2002; Eimer et al., 2003; Holmes et al., 2003, 2005; Ashley et al., 2004; Santesso et al., 2008). Because the N170 can be modulated by emotional expressions, and because its latency and scalp topography can resemble the vMMN to facial expressions, differentiating these two components is difficult. This is especially true in vMMN studies in which emotional faces have been used as deviant faces among neutral standard faces (Zhao and Li, 2006; Astikainen and Hietanen, 2009; Chang et al., 2010; Li et al., 2012) since the differential response could result from enhanced N170 responses to emotional vs. neutral faces.

Moreover, assuming that vMMN to emotional facial expressions could be separated from N170, there might be an additional confounding factor to consider. Namely, it is unclear whether such a differential response (seemingly similar to vMMN) reflects a true mismatch response that is, a response indicating regularity violation. The other possibility is that the differential response reflects, solely or in part, varying levels of sensitivity to different facial emotions. A few recent studies have elucidated this issue. In the study by Stefanics et al. (2012), regularity violations involved rare changes in emotional expressions (infrequent fearful face among happy faces and vice versa) of constantly changing facial identities. A rarely presented facial expression elicited differential ERPs relative to the same emotion when it was presented as a frequent one (i.e., happy standard vs. happy deviant face, fearful standard vs. fearful deviant face) at 70–120 ms latency for the fearful faces, and at 170–360 ms latency covering N170 and P2 components for both the fearful and happy faces. In the study by Kimura et al. (2011), an immediate repetition of an emotional expression was presented as a deviant stimulus violating the pattern of constantly changing (fearful and happy) emotions while the participants were attending to faces wearing eyeglasses. This stimulus condition elicited responses associated to the regularity violation at relatively long latencies: ~280 ms after the onset of the fearful faces and 350 ms after the onset of the happy faces. In both of these studies, the experimental paradigms allowed the analysis of vMMN by comparing the ERPs elicited by two identical pictures (e.g., fearful faces presented as a standard and as a deviant stimulus). Since differential ERPs, i.e., vMMN, to these physically identical pictures were found, confounding by emotional processing as such can be ruled out.

The existing findings of vMMN as an index of regularity violation in facial expression processing are, however, only from experiments which applied happy and fearful faces in the stimulus series, i.e., all the expression used in the experiments were emotional expressions (Kimura et al., 2011; Stefanics et al., 2012). vMMN as an index of regularity violation and its possible confounding by emotional expression encoding remains an open question in the case of expressive vs. neutral faces. In our previous study (Astikainen and Hietanen, 2009), neutral standard faces of constantly changing identities were presented, and the regularity violations were rarely presented fearful or happy faces. A differential response was elicited by the rare expressions at 150–180 ms latency, but this study left open the question of functional independency between N170 and vMMN as well as the question of the emotional confounding of the vMMN response. The same holds true for a study in which regularity of “neutral expression” was violated by happy and sad faces while changing “identities” (i.e., low-level visual features) of schematic faces were used (Chang et al., 2010).

In order to reveal the process underlying the differential responses to deviant emotional vs. neutral standard faces (regularity violation vs. encoding of emotional expression) and its independency from (emotion-modulated) N170, we recorded ERPs in two different conditions presented to two groups of adult humans. For one group, happy, fearful, and neutral faces were presented in random order and with an equal probability (*p* = 0.33 for each; equiprobable condition). For the other group, fearful and happy faces were rarely (*p* = 0.1 for both) and pseudo-randomly (at least two neutral faces were presented between the emotional faces) interspersed with the neutral ones (oddball condition). In both conditions, the facial identities changed from trial to trial requiring abstraction of the regularity in the facial expressions from among several low-level features. Independent component analysis (ICA) was applied to the data. ICA functions as a spatial filter for the ERP data, and the peak amplitudes of the components projected to the sensor space can be further used in statistical analysis.

We expected to find two separate components in the oddball condition: emotion modulated N170 and vMMN. In the equiprobable condition, we expected to observe only emotion-modulated N170. It was also possible that no differential response would be found in the equiprobable condition if N170 was not sensitive to the emotional facial expressions in the present stimulus presentation condition.

## Methods

### Participants

Twenty native Finnish-speaking volunteers participated in the study. For half of the participants, the stimuli were presented in the oddball condition whereas the other half viewed the stimuli in the equiprobable condition (see below). Both groups comprised two male and eight female participants. In the “oddball” group, the age range was 19–35 years and mean age 23.9 years (median 23.5). In the “equiprobable” group, the age range was 20–42 years with a mean age of 24.6 years (median 21.5). All the participants were right-handed and had self-reported normal hearing and vision (corrected with eyeglasses if necessary), and no diagnosed neurological or psychiatric disorders. A written informed consent was obtained from the participants before their participation. The experiment was undertaken in accordance with the Declaration of Helsinki. The ethical committee of the University of Jyväskylä approved the research protocol.

### Procedure

During the experiment, the participants sat in a comfortable chair in a dimly-lit room. The participants were instructed to attend to a recording of a radio play. The play was presented via a loudspeaker placed at about 50 cm above the participant's head where the volume of the recording equaled that of a normal speaking voice. Visual stimuli were presented on a computer screen (Eizo Flexscan, 17 inch CRT display) approximately one meter away from the participant. The participants were monitored during the recordings via a video camera positioned on top of the screen. The participants were asked to fix their gaze at a cross in the middle of a screen. The participants were informed that the visual stimuli would be faces and they were instructed to concentrate on the radio play and pay no attention to the faces.

### Stimuli

The visual stimuli were pictures of faces of four different models (male actors PE and JJ, female actors MF and NR) from Pictures of Facial Affect (Ekman and Friesen, 1976). Pictures of a neutral, fearful and happy expression from each model were used. The stimulus presentation was controlled with E-Prime software (Psychology Software Tools, Inc., Sharpsburg, MD, USA).

The pictures of faces, occupying an area of 4 × 5 ° of visual angle were presented at fixation for 200 ms. The stimulus-onset asynchrony (SOA) was 700 ms. The faces were presented in two different conditions (a between-subjects variable). In a modified oddball condition, two different deviant stimulus types, fearful faces and happy faces, were infrequently interspersed between frequently presented neutral standard faces. Standards and deviants were presented pseudo-randomly with the restriction that no less than two standards would occur between consecutive deviants. Among the 1600 stimuli were 160 happy faces (*p* = 0.1) and 160 fearful faces (*p* = 0.1). In the equiprobable condition, the stimulus presentation was otherwise the same, except that all the expressions were presented pseudo-randomly (there were no immediate repetitions of stimuli from the same emotion category), and with equal probability (*p* = 0.33). The number of stimuli in each emotion category was the same as the number of deviants in the oddball condition that is, 160 stimuli in each emotion category were presented. In both stimulus presentation conditions the facial identity in the pictures changed from trial to trial.

### Electroencephalography recording

Electroencephalogram was recorded with Brain Vision Recorder software (Brain Products GmbH, Munich, Germany) at Fz, F3, F4, Cz, C3, C4, Pz, P3, P4, P7, P8, Oz, O1, and O2 according to the international 10–20 system. An average reference was applied. Eye movements and blinks were measured from bipolar electrodes, one placed above the left eye and the other lateral to the right orbit. The signals from the electrodes were amplified, sampled at a rate of 1000 Hz, digitally band pass filtered from 0.05 to 100 Hz, and stored on a computer disk.

### Data analysis

**Figure 1:**
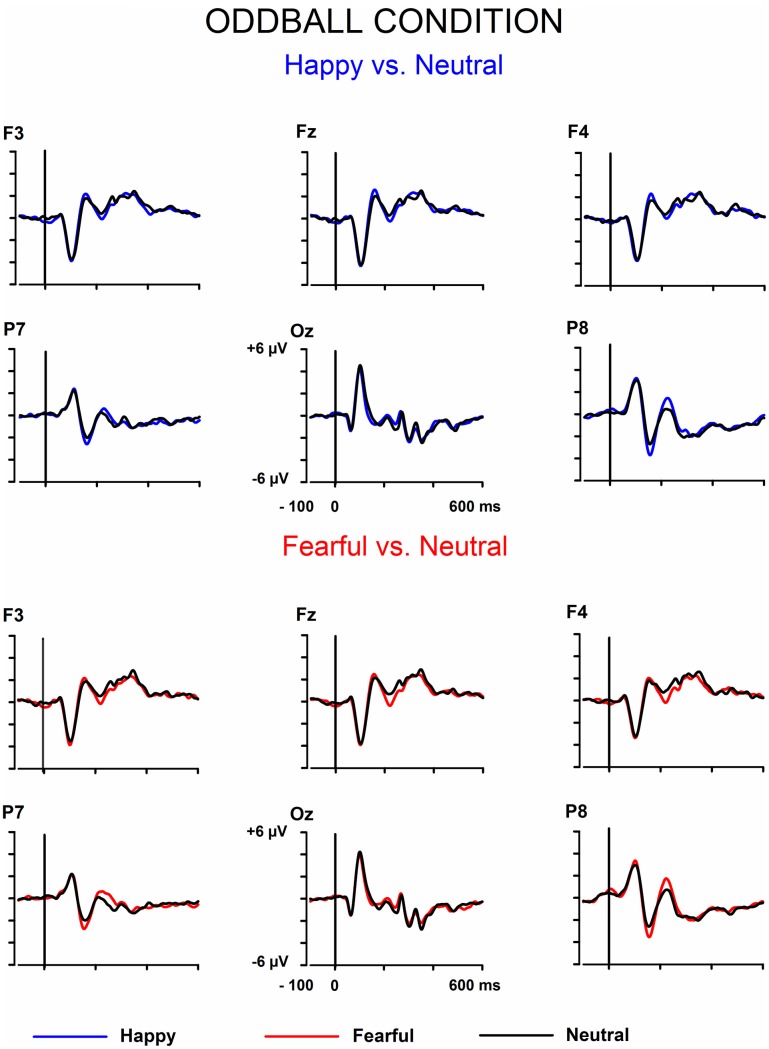
**Grand-averaged ERPs in the oddball condition**. Raw ERPs to happy and fearful deviant faces and to the neutral standard faces immediately preceding them. Stimulus onset at time 0.

**Figure 2:**
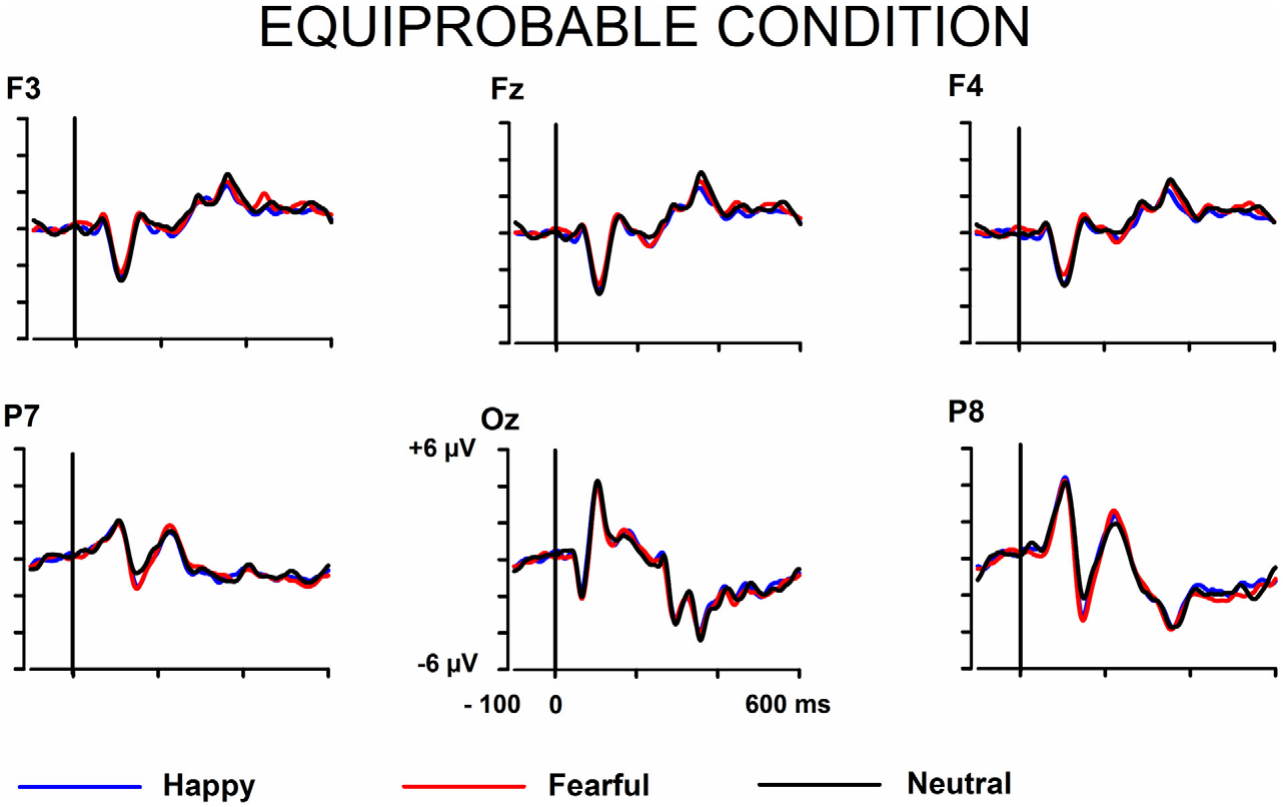
**Grand-averaged ERPs in the equiprobable condition**. Raw ERPs to happy, fearful, and neutral faces. Stimulus onset at time 0.

The signals from the electrodes were filtered (Butterworth zero phase filter: 0.1–30 Hz, 24 dB/octave roll-off) and 700-ms stimulus-locked segments were extracted (from −100 to +600 ms). Segments with signal amplitudes beyond the range between −100 and 100 μ V in any recording channel, including the EOG channel, were omitted from further analysis. The segments were corrected by their baseline values (mean amplitude during the 100-ms pre-stimulus period). In the equiprobable condition, all the responses left after the artifact rejection were averaged for each participant. On average, 136 trials for the fearful (min = 107, max = 155, median = 141), 135 trials for the happy (min = 96, max = 154, median = 142), and 135 trials for the neutral expression (min = 105, max = 155, median = 139) were available. In the oddball condition, only responses to standards immediately preceding the deviants were averaged. This procedure allows the same number of segments, and thus a similar signal-to-noise ratio, for both standards and deviants. On average, the number of analyzed trials for the fearful and happy deviants and the neutral standards immediately preceding them was 138 (fear trials: min = 85, max = 160, median = 149; happy trials: min = 78, max = 158, median = 153). Figure 1 depicts the ERPs to the happy, fearful, and neutral faces in the oddball condition and Figure 2 to those in the equiprobable condition.

**Figure 3:**
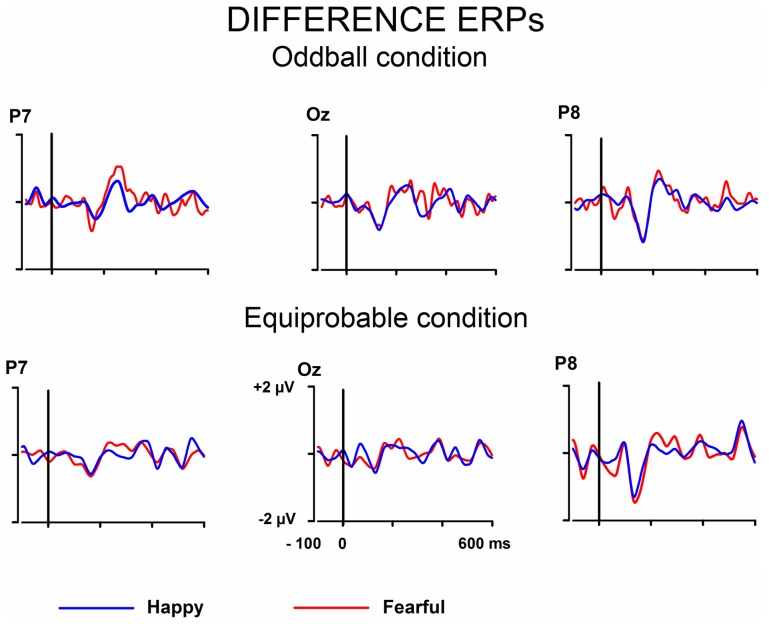
**Grand-averaged differential ERPs (emotional minus neutral face)**. Raw ERPs.

Next, differential ERPs (expressive minus neutral face responses) were calculated separately for the fearful and happy faces. By this way, the brain activities common to the emotional and neutral faces were removed. The differential ERPs were processed by an approach including wavelet filter and ICA. The approach and the benefits of it has been thoroughly described by Cong et al. (2011a,b, 2012). In this approach, ICA is applied to the averaged ERPs (see also Makeig et al., 1997; Vigario and Oja, 2008; Kalyakin et al., 2008, 2009; Cong et al., 2011a,b). This is different from the commonly used application of ICA on the concatenated single-trial EEG data (for a N170 study, see e.g., Desjardins and Segalowitz, 2013). Briefly, the method was as follows. Wavelet filter was performed on the difference wave. Ten levels were set to decompose the signal through the reversal biorthogonal wavelet with the order of 6.8, and coefficients at levels 5–8 were chosen for the reconstruction. The wavelet filter was selected so that it could be assumed to remove sensor noise and frequencies irrelevant for the studied components (Cong et al., 2011b, 2012). This has been found to be advantageous in the following ICA decomposition (Cong et al., 2011a,b). Figure 3 shows the wavelet-filtered differential responses in both stimulus presentation conditions.

The filtered and averaged differential ERPs (responses to fearful and happy faces minus responses to neutral standard faces) were next decomposed by ICASSO software for ICA (Himberg et al., 2004). The unmixing matrix was randomly initialized 100 times, and FastICA with the hyperbolic tangent function (Hyvärinen, 1999) was run 100 times for each setting to extract 14 components each time. The 1400 components obtained in the 100 runs were then clustered into 14 groups using agglomerative hierarchical clustering with the average-linkage criterion. Finally, the centroid of each cluster was sought and was regarded as one component by ICASSO (Himberg et al., 2004). The stability of the ICA decomposition was satisfactory: the mean of the index of quality (Iq) of the 560 ICA components (2 by 2 by 10 by 14) was 0.90 (standard deviation = 0.10, min = 0.45, max = 0.99, and median = 0.94). This index is for the interpretation of the stability of decomposition for each ICA component. If the Iq is close to “1,” multiple runs of ICA decomposition give similar results, which means that ICA composition is stable. Otherwise, if the Iq is close to “0,” multiple runs of ICA decomposition give very different results.

After the estimation of the independent components by ICASSO, the desired components were chosen based on the peak latency between 100 and 200 ms and subsequent evaluation of the component's scalp topography (posterior negativity) when the component was projected back to the electrodes. This projection was to correct the inherent polarity and variance indeterminacy of ICA (Makeig et al., 1999). It also allows performing conventional statistical analysis on peak amplitudes to reveal the experimental effects. Two components with peak latencies of ~130 and 170 ms after stimulus onset were found for each participant in both conditions.

The peak amplitudes of the electrode-field projection of ICA components were submitted to repeated measures multivariate analysis of variance (MANOVA) with within-subjects factors of Expression (fearful vs. happy) and Electrode (Fz, F3, F4, Pz, P7, P8, Oz), and with a between-subjects factor Condition (oddball condition vs. equiprobable condition). Channel selection was based on visual inspection of the grand averaged scalp topography maps and previous findings for N170 (e.g., Blau et al., 2007) and vMMN to facial emotions (e.g., Astikainen and Hietanen, 2009; Stefanics et al., 2012). *P*-values smaller than 0.05 were considered as significant. *T*-tests were two-tailed, and their test-values are reported whenever the *p*-value is smaller than 0.055. Partial eta squared (η^2^_p_) presents effect size estimates for MANOVA.

## Results

Figures 1, 2 show the raw grand-averaged ERPs for the oddball and equiprobable groups. In Figure 3, the grand-averaged differential responses (emotional minus neutral) for both conditions are presented. In these figures, differential responses to both emotional expressions and in both stimulus presentation conditions can be observed in the lateral parietal and occipital electrodes in the latency range under inspection (100–200 ms post stimulus).

**Figure 4:**
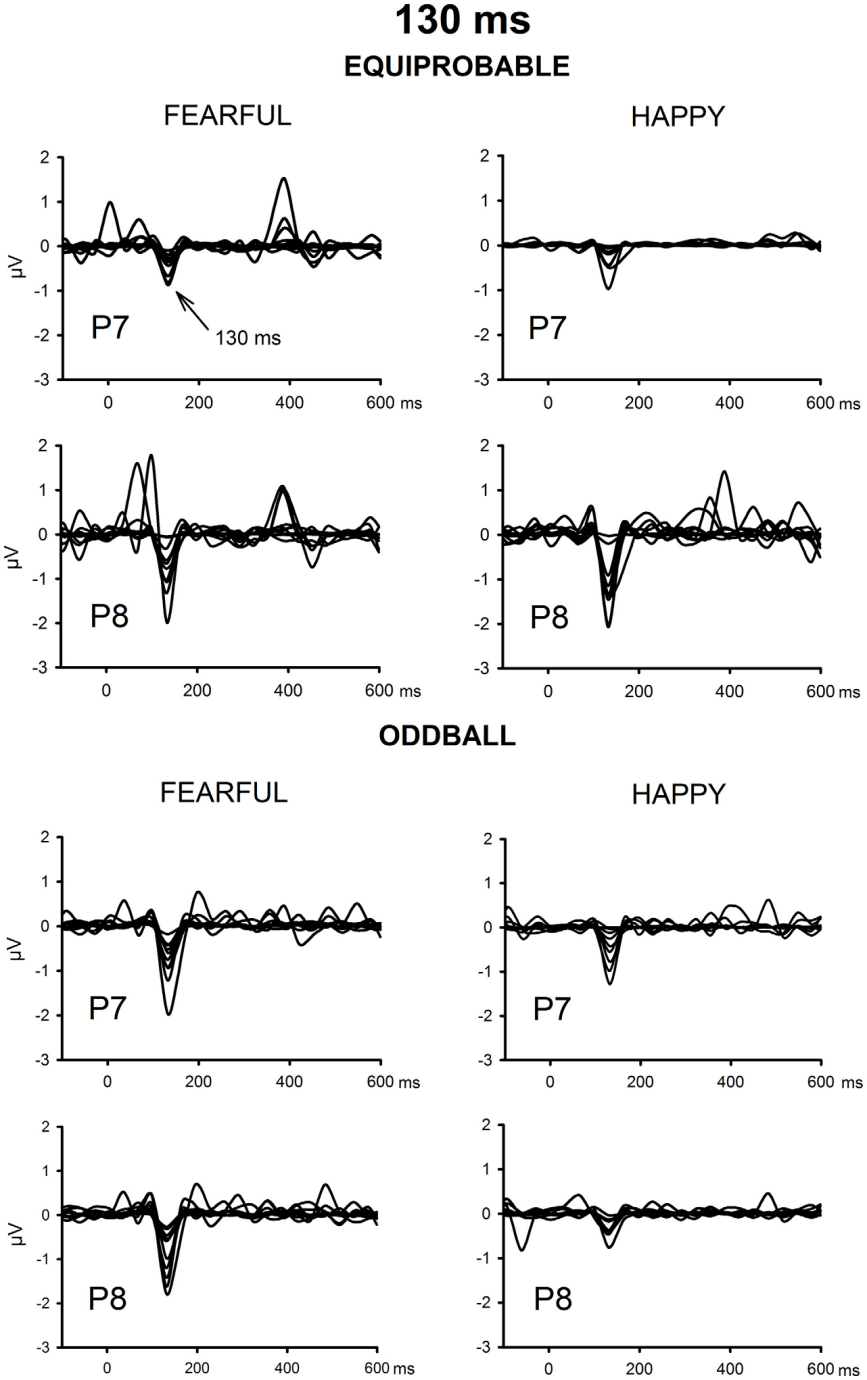
**Back-projected 130-ms components for each participant**. Differential response waveforms (emotional minus neutral) at P7 and P8 electrodes are drawn separately for fearful and happy faces and for oddball condition and equiprobable condition groups.

**Figure 5:**
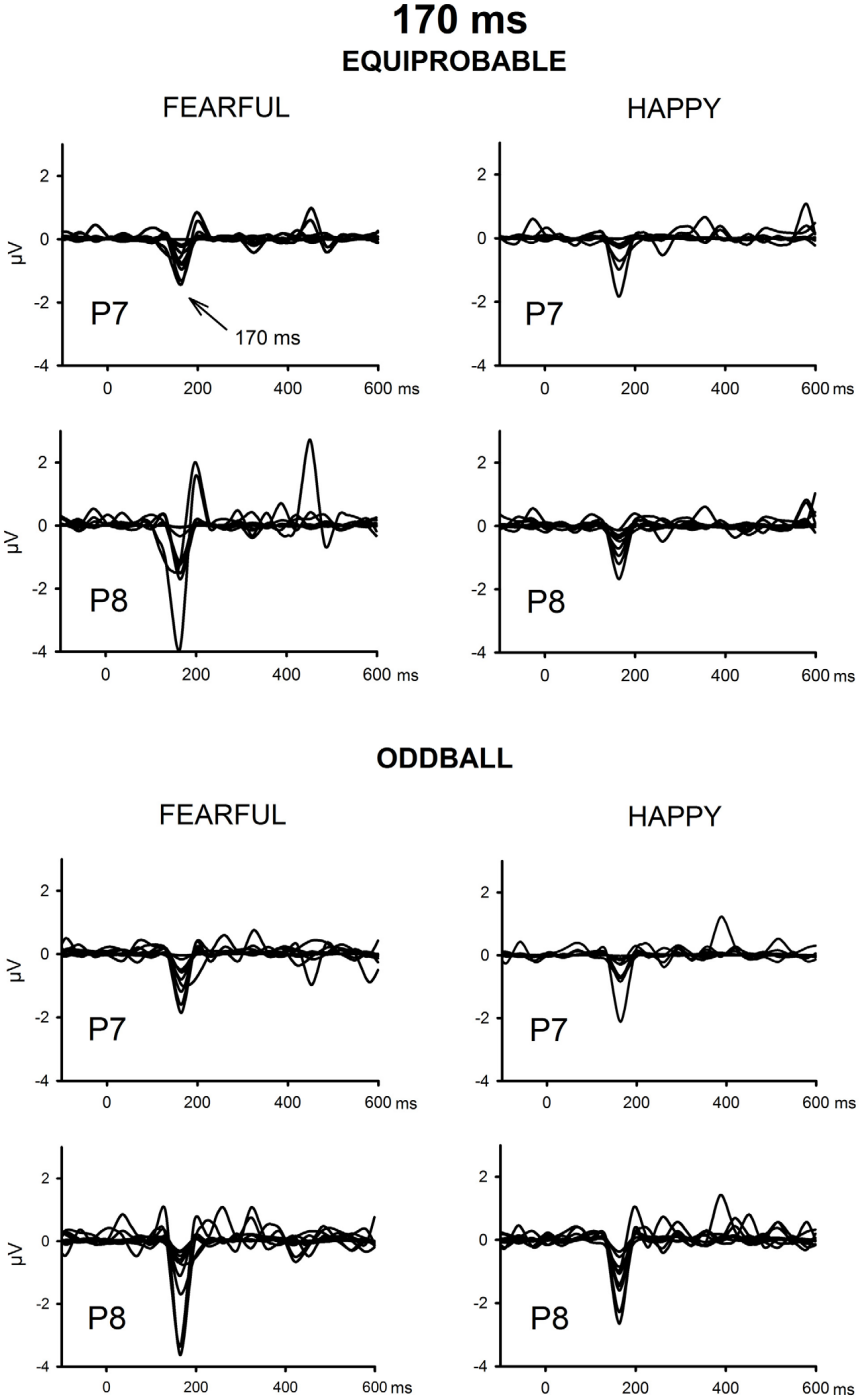
**Back-projected 170-ms components for each participant**. Differential response waveforms (emotional minus neutral) at P7 and P8 electrodes are drawn separately for fearful and happy faces and for oddball condition and equiprobable condition groups.

The ICA decomposition showed two separate components at the relevant latency range for both the oddball and equiprobable condition. The earlier, henceforth 130-ms component, had a mean latency of 134 ms, and the later, henceforth 170-ms component, had a mean latency of 165 ms in the posterior electrode sites. Figures 4, 5 illustrate the back-projected components in individual participants as waveforms at electrodes P7 and P8.

### 130-ms component

**Figure 6:**
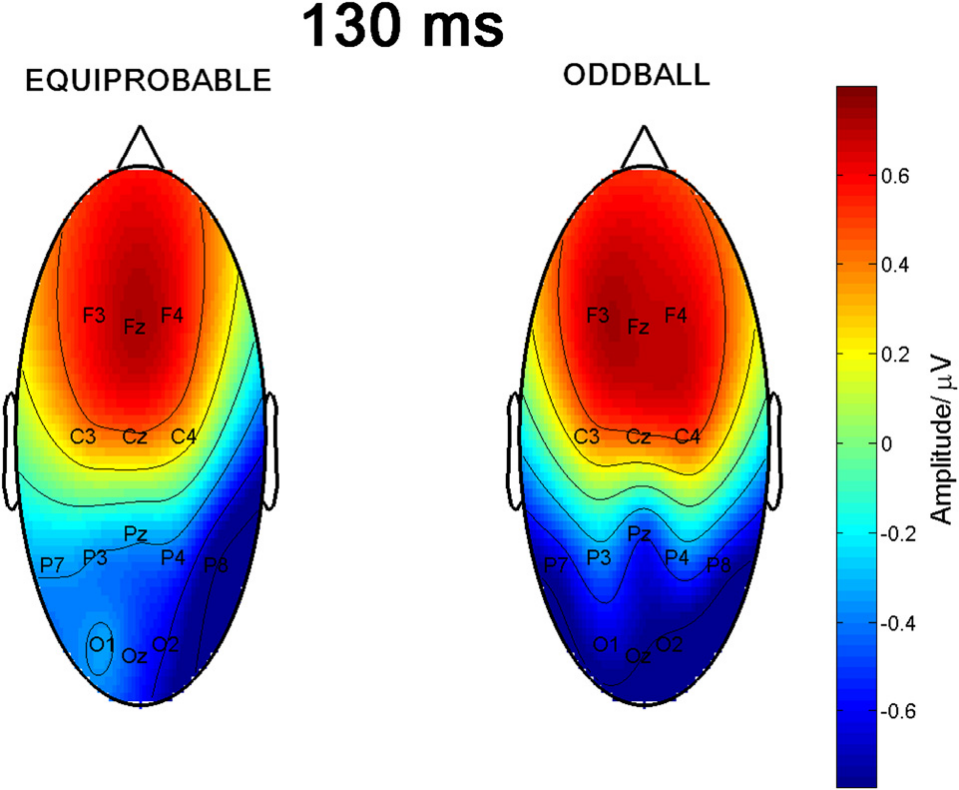
**Scalp potential maps of the 130-ms component back-projected to the electrodes**. Map for the equiprobable condition group on left and map for the oddball condition group on right.

Figure 6 shows the scalp potential maps for the 130-ms component back-projected to the electrodes in the oddball and in the equiprobable conditions.

A MANOVA revealed a significant main effect of Electrode, *F*_(6, 13)_ = 29.4, *p* < 0.0001, η^2^_p_ = 0.931, reflecting the positive polarity of the differential response in the frontal electrode sites and negative polarity in the posterior electrodes. Importantly, an Electrode × Condition interaction was found, *F*_(6, 13)_ = 5.00, *p* = 0.007, η^2^_p_ = 0.698, indicating a non-homogeneous scalp distribution in ERP amplitudes between the two conditions. The other main effects or other interaction effects were non-significant [Expression: *F*_(1, 18)_ = 0.45, *p* = 0.834, η^2^_p_ = 0.003; Expression × Condition: *F*_(1, 18)_ = 0.39, *p* = 0.846, η^2^_p_ = 0.002; Electrode × Expression: *F*_(6, 13)_ = 0.76, *p* = 0.617, η^2^_p_ = 0.259; Electrode × Expression × Condition: *F*_(6, 13)_ = 1.67, *p* = 0.206, η^2^_p_ = 0.435].

**Figure 7:**
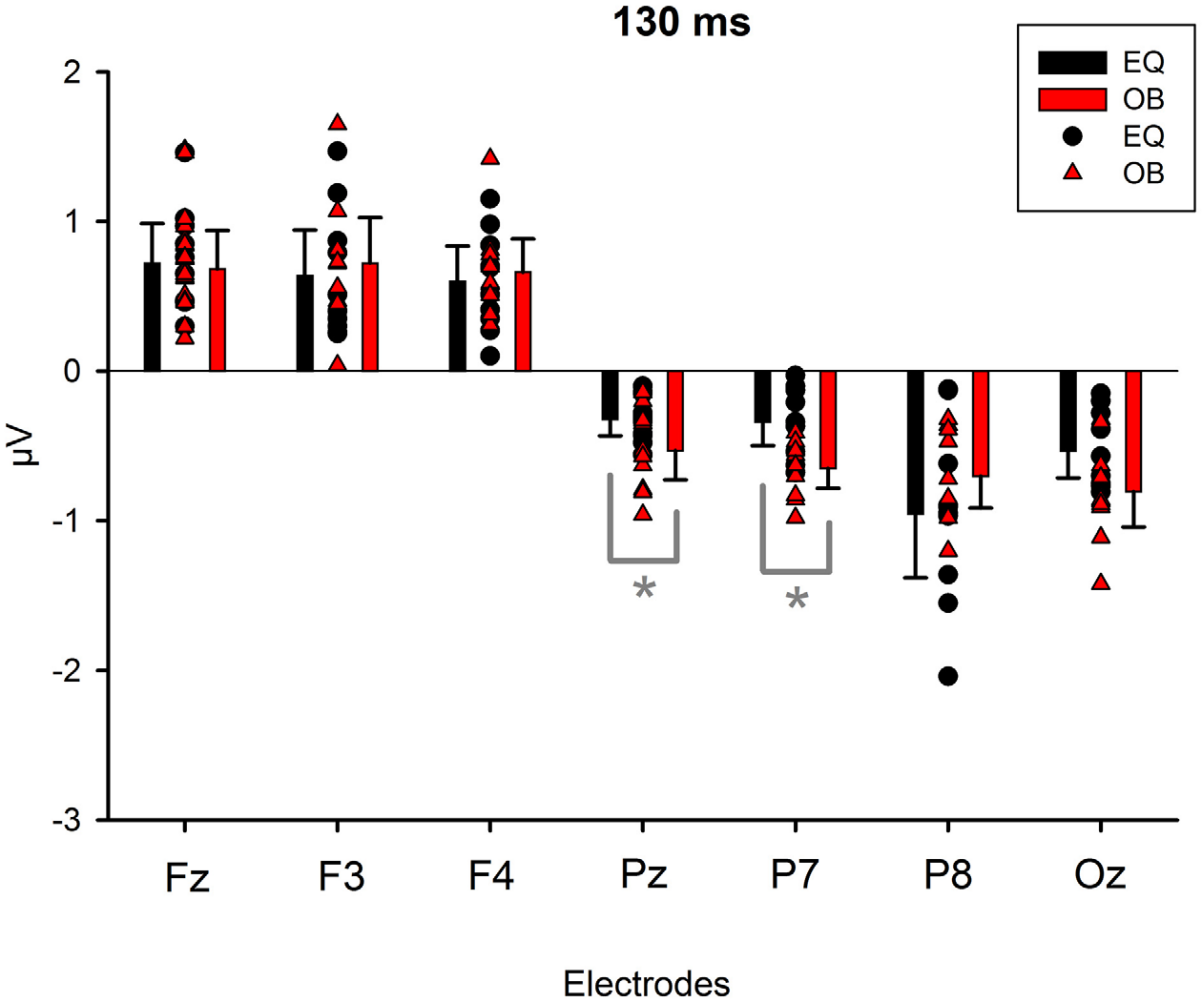
**Mean amplitude values (μV), confidence intervals, and scatterplots of the individual participants' values for each electrode in the equiprobable condition (EQ) and oddball condition (OB) for the 130-ms component (differential response; emotional minus neutral face)**. An asterisk (^*^) indicates a significant difference (*p* < 0.05) between conditions at P7 and Pz electrodes.

Because Expression showed no effect, subsequent *t*-tests with the mean amplitude values averaged over responses to fearful and happy faces were applied separately to data measured from each electrode in order to compare the responses between the stimulus presentation conditions. The stimulus presentation condition had a significant effect on differential responses (emotional minus neutral) at P7, *t*_(18)_ = 3.38, *p* = 0.011 (mean difference 0.31μV, 95% confidence interval 0.12–0.51 μV) and at Pz electrodes, *t*_(18)_ = 2.13, *p* = 0.047 (mean difference 0.21μV, 95% confidence interval 0.003–0.42 μV). There was also a marginally significant effect at Oz electrode, *t*_(18)_ = 2.07, *p* = 0.053 (mean difference 0.27 μV, 95% confidence interval 0.04–0.55 μV). For all these electrodes (P7, Pz, Oz), the difference-wave amplitudes were larger (more negative) in the oddball condition than in the equiprobable condition (Figure 7). For the other electrodes, no significant differences between the conditions were observed. Amplitude values differed from zero at all electrode sites and in both stimulus presentation conditions. Table 1 shows the *t*-values, *p*-values, mean differences, and 95% confidence intervals for the differential response amplitude values at each electrode tested against zero (one sample *t*-test).

**Table 1:** **130-ms component**.

**Electrode site/condition**	***t***	***p* (2-tailed)**	**Mean difference (μV)**	**95% Confidence interval of the difference**	
				**Lower**	**Upper**
Fz/OB	5.98	0.0001	0.68	0.42	0.94
Fz/EQ	6.24	0.0001	0.72	0.46	0.99
F3/OB	5.40	0.0001	0.72	0.42	1.03
F3/EQ	4.73	0.001	0.64	0.33	0.94
F4/OB	6.78	0.0001	0.66	0.44	0.88
F4/EQ	5.71	0.0001	0.60	0.36	0.84
Pz/OB	−6.12	0.0001	−0.53	−0.73	−0.34
Pz/EQ	−6.54	0.0001	−0.32	−0.43	−0.21
P7/OB	−11.08	0.0001	−0.65	−0.78	−0.52
P7/EQ	−4.75	0.001	−0.34	−0.50	−0.18
P8/OB	−7.38	0.0001	−0.70	−0.92	−0.49
P8/EQ	−5.08	0.001	−0.96	−1.38	−0.53
Oz/OB	−7.68	0.0001	−0.81	−1.05	−0.57
Oz/EQ	−6.65	0.0001	−0.53	−0.72	−0.35

One sample t-tests (tested against 0) for the emotional minus neutral differential responses. OB, oddball condition; EQ, equiprobable condition.

**Figure 8:**
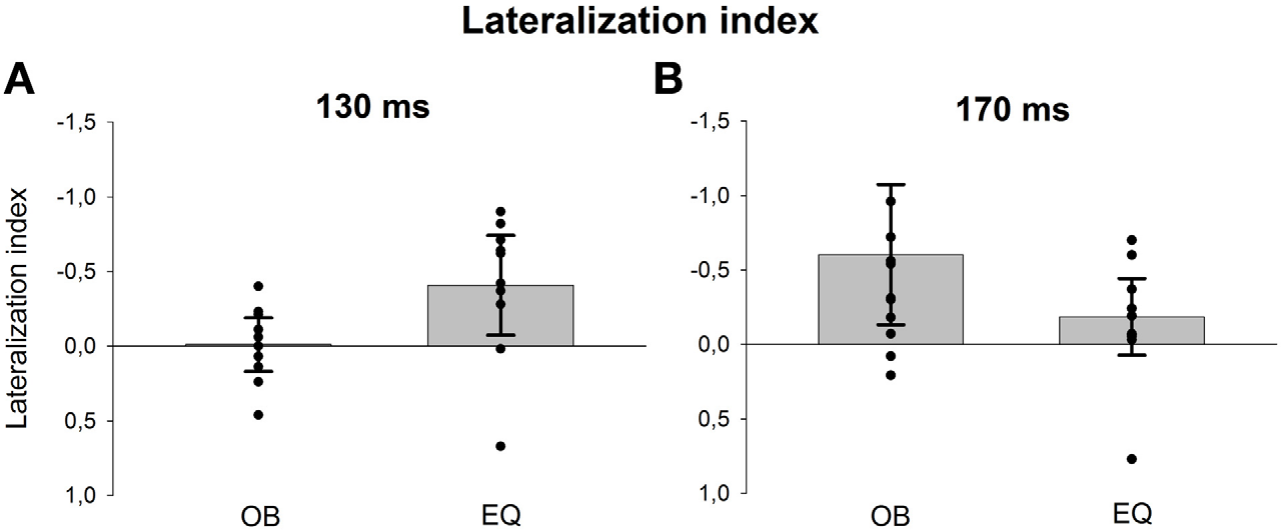
**Lateralization index for the 130-ms (A) and 170-ms (B) components separately for both conditions (oddball = OB and equiprobable = EQ)**. The values are calculated for the back-projected components' amplitudes from electrodes P7 (left) and P8 (right) as follows: (left – right)/(left + right). The bars represent the mean values in the group and the whiskers 95% confidence intervals. Individual participants' values are marked with filled circles.

Since visual inspection of the scalp topographies suggested that the lateral parietal activity was right dominant in the equiprobable condition, while no such lateralization existed in the oddball condition, Electrode × Condition effect was further studied. Pair-wise comparisons were applied for the amplitude values recorded at P7 and P8 separately for the conditions. The statistics conformed to visual observation showing that, in the equiprobable condition, amplitude values were larger in the right parietal electrode site than the left (i.e., P8 vs. P7), *t*_(9)_ = 3.19, *p* = 0.022 (Bonferroni corrected, mean difference 0.62 μV, 95% confidence interval 0.18–1.06 μV). In the oddball condition, no such difference was found, *t*_(9)_ = 0.48, *p* = 0.641 (mean difference 0.05 μV). Figure 8A depicts lateralization index for both conditions (oddball and equiprobable). Comparison of the lateralization indexes in the oddball and equiprobable conditions indicated a significant difference between the conditions, *t*_(18)_ = 2.36, *p* = 0.030, mean difference 0.40 μV, 95% confidence interval 0.04–0.75 μV).

### 170-ms component

**Figure 9:**
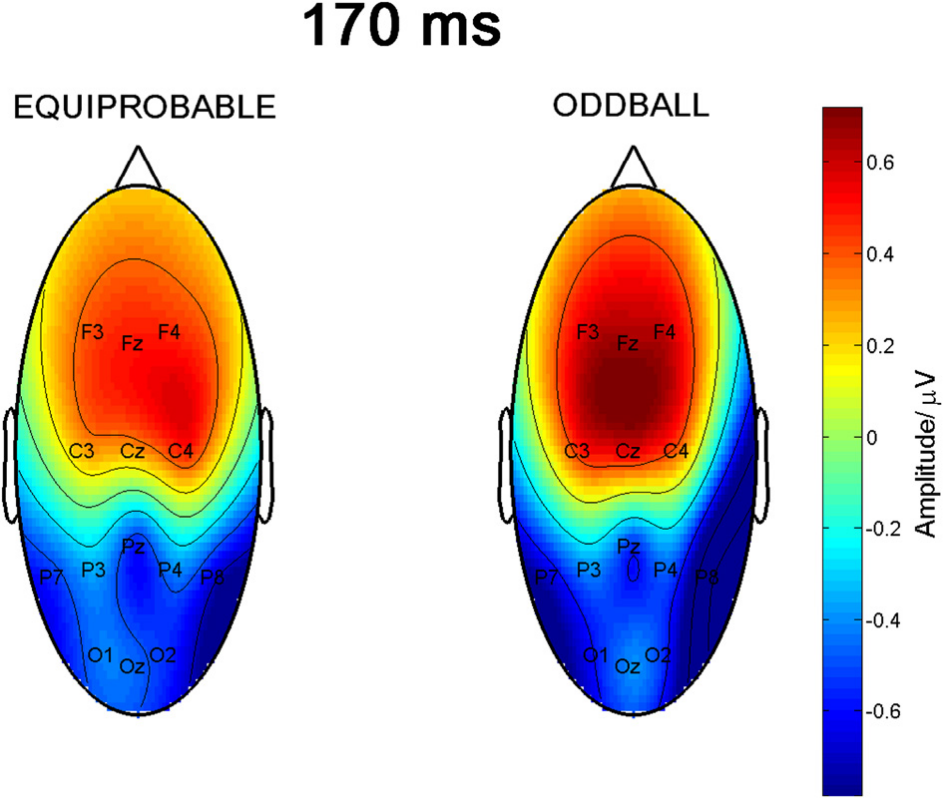
**Scalp potential maps of the 170-ms component back-projected to the electrodes**. Map for the equiprobable condition group on left and map for the oddball condition group on right.

**Figure 10:**
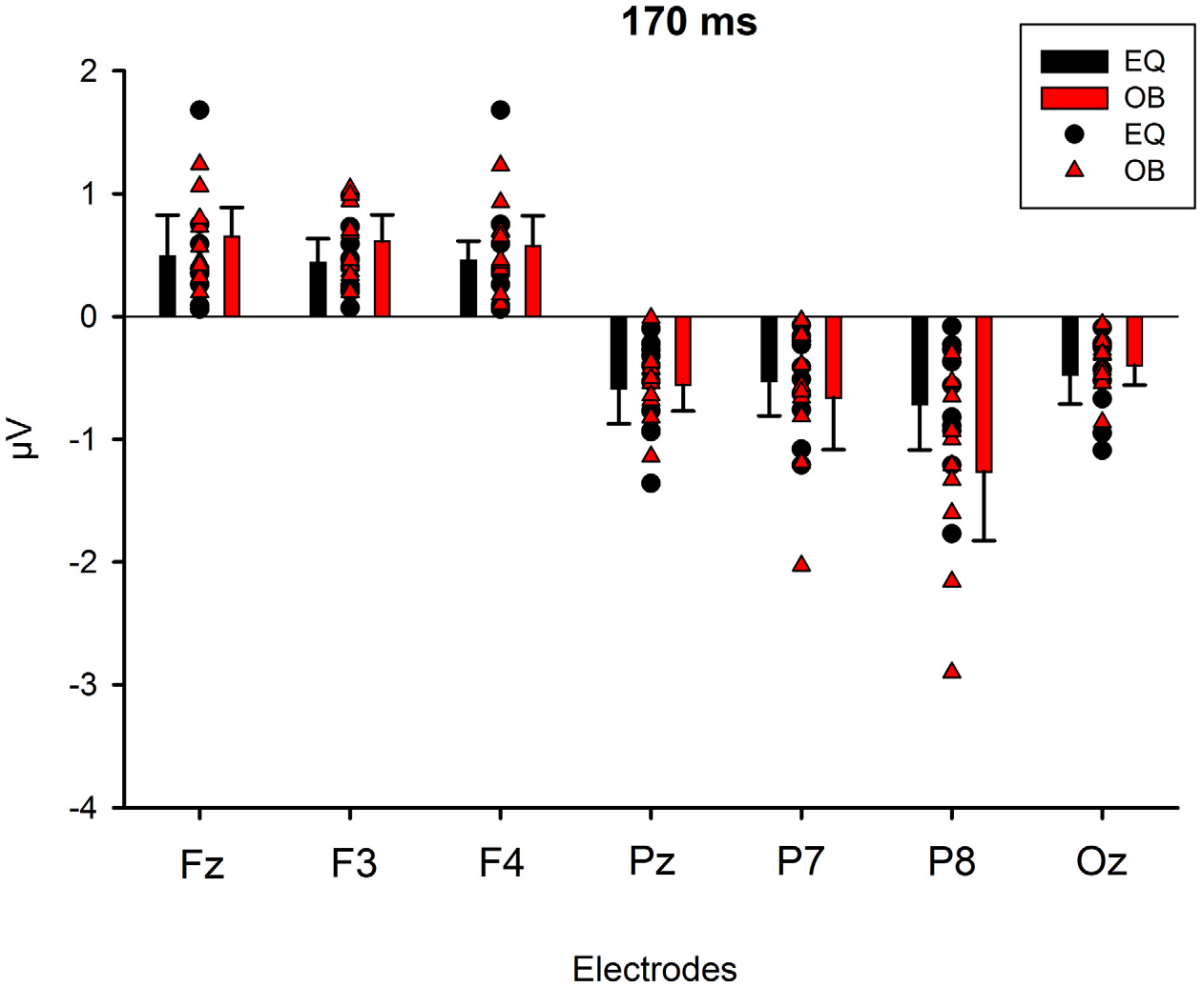
**Mean amplitude values (μV) and confidence intervals, and scatterplots of the individual participants' values for each electrode in the equiprobable condition (EQ) and oddball condition (OB) for the 170-ms component (differential response; emotional minus neutral face)**. No significant differences between conditions were found.

Figure 9 shows the scalp potential maps for the back-projected 170-ms component. A MANOVA indicated a main effect for electrode, *F*_(6, 13)_ = 14.11, *p* < 0.0001, η^2^_p_ = 0.867. No other main effects or any of the interaction effects were significant (Expression: *F*_(1, 18)_ = 0.35, *p* = 0.563, η^2^_p_ = 0.019; Electrode × Condition: *F*_(6, 13)_ = 0.68, *p* = 0.669, η^2^_p_ = 0.239; Expression × Condition: *F*_(1,18)_ = 0.66, *p* = 0.800, η^2^_p_ = 0.004; Electrode × Expression: *F*_(6, 13)_ = 0.56, *p* = 0.752, η^2^_p_ = 0.207; Electrode × Expression × Condition: *F*_(6, 13)_ = 0.34, *p* = 0.901, η^2^_p_ = 0.137). The effect for electrode resulted from the amplitudes being of positive polarity in the anterior electrodes and of negative polarity in the posterior electrodes (Figures 9, 10). Amplitude values averaged for the fearful and happy expressions differed from zero at all the electrode sites and in both stimulus presentation conditions. Table 2 shows the *t*-values, *p*-values, mean differences, and 95% confidence intervals for the differential response amplitude values at each electrode tested against zero (one sample *t*-test).

**Table 2:** **170-ms component**.

**Electrode site/condition**	***T***	***p* (2-tailed)**	**Mean difference (μV)**	**95% Confidence interval of the difference**	
				**Lower**	**Upper**
Fz/OB	6.32	0.0001	0.65	0.42	0.89
Fz/EQ	3.35	0.009	0.49	0.16	0.82
F3/OB	6.50	0.0001	0.61	0.40	0.83
F3/EQ	5.02	0.001	0.44	0.24	0.63
F4/OB	5.36	0.0001	0.58	0.33	0.82
F4/EQ	6.38	0.0001	0.45	0.29	0.62
Pz/OB	−5.91	0.0001	−0.56	−0.77	−0.34
Pz/EQ	−4.58	0.001	−0.58	−0.87	−0.30
P7/OB	−3.52	0.007	−0.66	−1.08	−0.24
P7/EQ	−4.24	0.002	−0.53	−0.81	−0.25
P8/OB	−5.05	0.001	−1.26	−1.83	−0.70
P8/EQ	−4.36	0.002	−0.71	−1.08	−0.34
Oz/OB	−5.65	0.0001	−0.40	−0.56	−0.24
Oz/EQ	−4.40	0.002	−0.47	−0.71	−0.23

One sample t-tests (tested against 0) for the emotional minus neutral differential responses. OB, oddball condition, EQ, equiprobable condition.

Figure 8B shows lateralization index for both conditions (oddball and equiprobable). No statistically significant difference was found in the lateralization indexes between the conditions, *t*_(18)_ = 1.06, *p* = 0.304.

## Discussion

We presented two groups of adults with a series of pictures of faces: for one group the faces were presented in an oddball condition, for the other group the faces were presented in an equiprobable condition. Facial identities changed on a trial-by-trial basis. For the oddball condition group, most of the faces expressed neutral emotion, with rare happy and fearful faces randomly violating this regularity. For the equiprobable condition group, neutral, happy, and fearful faces were presented with equal probability and formed no regularity in the stimulus series.

Differential responses to emotional expressions (fearful–neutral and happy–neutral) were calculated, wavelet filtering was applied to the averaged data in order to increase the signal-to-noise ratio and the independent components were extracted by the open-source ICA software, ICASSO (Himberg et al., 2004). We found two separate components for the emotional faces in both the oddball and equiprobable conditions: one at the latency of ~130 ms and the other at the latency of ~170 ms after stimulus onset.

The 170-ms component conforms to the face-sensitive N170 response. The scalp topography of the component extracted from the differential response included a lateral occipito-parietal negativity and frontal positivity, and was thus similar to the topography previously reported for N170 itself (e.g., Bentin et al., 1996; Ashley et al., 2004; Williams et al., 2006; Blau et al., 2007). Also Blau et al. (2007) have found that subtracting responses to task-irrelevant fearful faces from those to neutral faces provided a topography that was highly similar to the topography of N170. The frontal positivity was most likely a so called vertex positive potential (VPP) known to be elicited by the same brain generators as N170 (Joyce and Rossion, 2005). Also the present data analysis based on ICA method supports this view.

The 170-ms component did not, as expected, differ between the stimulus presentation conditions, but differentiated between the emotional and neutral faces. Emotional modulation of N170 has also been reported in several other studies (Batty and Taylor, 2003; Eger et al., 2003; Caharel et al., 2005; Williams et al., 2006; Blau et al., 2007; Leppänen et al., 2007; Schyns et al., 2007; Japee et al., 2009; Vlamings et al., 2009; Wronka and Walentowska, 2011). Since there are also studies in which no N170 modulation for emotional expressions has been found (Eimer and Holmes, 2002; Eimer et al., 2003; Holmes et al., 2003, 2005; Ashley et al., 2004; Susac et al., 2004; Santesso et al., 2008), future studies should explore the factors influencing this modulation. These could be related to several methodological choices, for example, to the behavioral tasks the participants are asked to perform during stimulus presentation. Recently, the location of the reference electrode has also been suggested to have an effect on the N170 modulation by emotional expression (Rellecke et al., 2013).

The finding of a 130-ms component in both conditions was unexpected. It was observed as enhanced parieto-occipital negativity and frontal positivity to the emotional faces in comparison to neutral faces in both conditions. Importantly, however, differences in topography between the conditions were observed: the topography was bilateral over the lateral parietal sites in the oddball condition while it was more right-dominant in the equiprobable condition. The bilateral posterior topography of the component conforms to vMMN to facial expressions (Astikainen and Hietanen, 2009; Stefanics et al., 2012). Also, the observed frontal positivity in the present data replicates our previous findings of the vMMN topography to emotional faces (Astikainen and Hietanen, 2009). The current data suggest that this differential response is not due to detection of the regularity violation, but more generally related to emotional processing since it was elicited also in the equiprobable condition. Indeed, in some previous studies investigating ERPs to facial expressions, but not applying the oddball condition, a frontal positivity to emotional expressions relative to neutral ones at a latency corresponding to that observed in the present study has been found (Eimer and Holmes, 2002; Kiss and Eimer, 2008). The latency of the 130-ms component is in line with the earliest differential responses to changes in facial expressions (Susac et al., 2004, 2010; Astikainen and Hietanen, 2009; Chang et al., 2010; Stefanics et al., 2012). The fact that elicitation of the 130-ms component was also observed in the equiprobable condition suggests that it reflects both the detection of the regularity violations and encoding of the emotional information in the faces. This finding calls for appropriate control conditions, such as an equiprobable condition, in future studies of vMMN to facial expressions.

In accordance with our previous finding of vMMN in a similar experimental paradigm as the oddball condition applied here (Astikainen and Hietanen, 2009), the 130-ms component showed no difference between fearful and happy expressions in either condition (oddball or equiprobable). Also in a study in which schematic faces were applied, sad and happy faces as rare changes among neutral standard faces elicited equally large amplitudes (Chang et al., 2010). On the other hand, a so called “negativity bias” in response latencies has been reported in two previous vMMN studies using facial expressions (Kimura et al., 2011; Stefanics et al., 2012). In these studies, fearful faces as deviants elicited differential responses in clearly earlier latency ranges than happy faces; for example, the differential response found in the 70–120-ms latency range to fearful faces was absent for happy faces (Stefanics et al., 2012). In the present study, the fearful and happy deviants elicited the same components (130− and 170-ms component) and they were also similar in their latencies. The lack of a negative bias in our study is not, however, in conflict with the previous findings by Kimura et al. (2011) and Stefanics et al. (2012), hence we only analyzed the components in the relevant latency range for N170 (100–200 ms post stimulus). Inspection of the components extracted by ICA for the entire post stimulus time period might have revealed emotion-specific components in the earlier and later latency ranges as well.

There are some limitations in the present study. First, the emotional expressions were not presented with the same probability in the oddball and equiprobable condition (*p* = 0.1 and *p* = 0.33, respectively). It is thus possible that it was not solely the differences in cognitive expectation (present only in the oddball condition in which the high probability of the neutral standard faces formed it), but also the differences in the probability of the emotional expressions as such that could have induced the between-condition effects. In the future, one should investigate whether the response amplitude of vMMN or N170 to emotional faces is influenced by the presentation probability within the stimulus sequence. Second, the study was conducted with a limited sample size. Future studies should aim to replicate the findings with a larger number of participants. Third, the current study is based on EEG data recorded with a montage of 14 electrodes. More sensors could have allowed for example estimation of the locations of sources (for a magnetoencephalography study of facial processing see, Smith et al., 2009). Finally, the present study does not reveal to which specific diagnostic features in faces the found components are responses to (see e.g., Schyns et al., 2009). However, in the present study, several different facial identities in the pictures were applied and there were no immediate repetitions in them. Our results might thus reflect abstraction of emotion-related features among several changing low-level features.

In sum, we found two separate components in the 100–200-ms latency range for changes in emotional expressions. The component peaking at ~170 ms post stimulus showed no difference between the stimulus presentation conditions and it was identified as the face-sensitive N170 response. A component peaking at 130 ms post stimulus was different in its scalp topography in the oddball and the equiprobable conditions, i.e., when the presented face violates the regularity formed by the standard faces in comparison to the condition in which no regularity is present. Future studies of vMMN to facial expressions should apply relevant control conditions to avoid the confounding effect of the encoding of emotional expressions as such.

### Conflict of interest statement

The authors declare that the research was conducted in the absence of any commercial or financial relationships that could be construed as a potential conflict of interest.

## Supplementary material

A data sample and Matlab scripts for running the data processing including wavelet filtering and ICA can be found online at: http://www.frontiersin.org/Human_Neuroscience/10.3389/fnhum.2013.00557/abstract
